# Supplementary material for: Ligand-induced conformational selection predicts the selectivity of cysteine protease inhibitors
Source: PLoS One. 2019 Dec 19;14(12):e0222055. doi: 10.1371/journal.pone.0222055 (PMC6922342; doi:10.1371/journal.pone.0222055)
Supplement: S1 Table — A, N and C are defined as the set of bins occupied by Apo, noncovalent and covalent simulations and ∩ the intersection of set. (PDF) [file pone.0222055.s001.pdf]

Table S 1 - Auxiliary metrics derived from two first principal components obtained for system all systems studied. A, N and C are defined as the set of bins occupied by Apo, noncovalent and covalent simulations and ∩ the intersection of set.

Metrics / Complex	|A|	|N|	|C|	|A∩N|	|N∩C|	|A∩C|
CatK-ICK	376	213	186	206	117	136
CatK-ICL	236	152	181	93	125	132
CatK-ICR	320	193	168	181	155	149
CatK-IKR	354	235	188	227	156	156
CatL-ICK	509	399	185	345	101	152
CatL-ICL	366	138	147	138	121	147
CatL-ICR	405	225	194	210	84	168
CatL-IKR	472	394	262	383	141	199
Cruz-BCR	1194	496	532	451	0	458
Cruz-ICK	621	473	318	415	197	187
Cruz-ICL	498	350	254	341	250	248
Cruz-ICR	689	409	329	368	263	281
Cruz-IKR	770	447	293	371	210	146
Cruz-n409	1232	461	543	408	412	500
Cruz-n544	1251	641	577	637	339	437
Cruz-n568	1216	733	675	689	278	428
Cruz-n569	1046	841	549	725	219	213
