# Supplementary material for: Ligand-induced conformational selection predicts the selectivity of cysteine protease inhibitors
Source: PLoS One. 2019 Dec 19;14(12):e0222055. doi: 10.1371/journal.pone.0222055 (PMC6922342; doi:10.1371/journal.pone.0222055)

Figure S 5 - Distance between BCR nitrile and sulfur from Cys25 residue (first column) e RMSD of the ligand (second column) complexed with cruzain. Black vertical bars delimite the replicates. Black and red lines represents respectively Round 1 and 2 simulations.

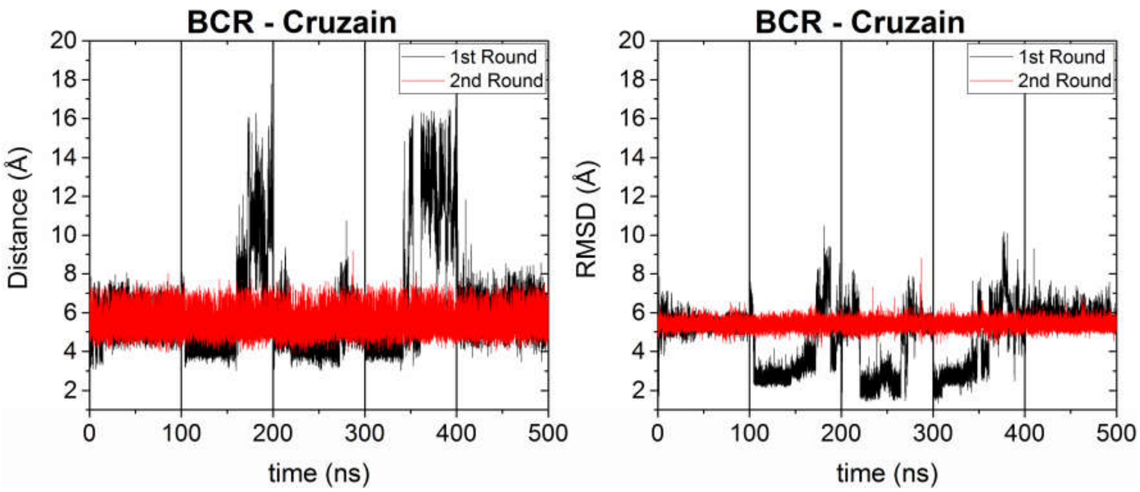

Supplement: S5 Fig — Black vertical bars delimit the replicates. Black and red lines represents respectively Round 1 and 2 simulations. (PDF) [file pone.0222055.s006.pdf]
