# Supplementary material for: Ligand-induced conformational selection predicts the selectivity of cysteine protease inhibitors
Source: PLoS One. 2019 Dec 19;14(12):e0222055. doi: 10.1371/journal.pone.0222055 (PMC6922342; doi:10.1371/journal.pone.0222055)
Supplement: S6 Fig — Black vertical bars delimit the replicates. Black and red lines represent respectively Round 1 and 2 simulations. The rows are respectively Neq0409, Neq0544, Neq0569, Neq0568. (PDF) [file pone.0222055.s007.pdf]

Figure S 6 - Distance (first column) between ligand nitrile and sulfur from Cys25 residue and RMSD of ligand (second column) complexed with cruzain. Black vertical bars delimite the replicates. Black and red lines represent respectively Round 1 and 2 simulations. The rows are respectively Neq0409, Neq0544, Neq0569, Neq0568.

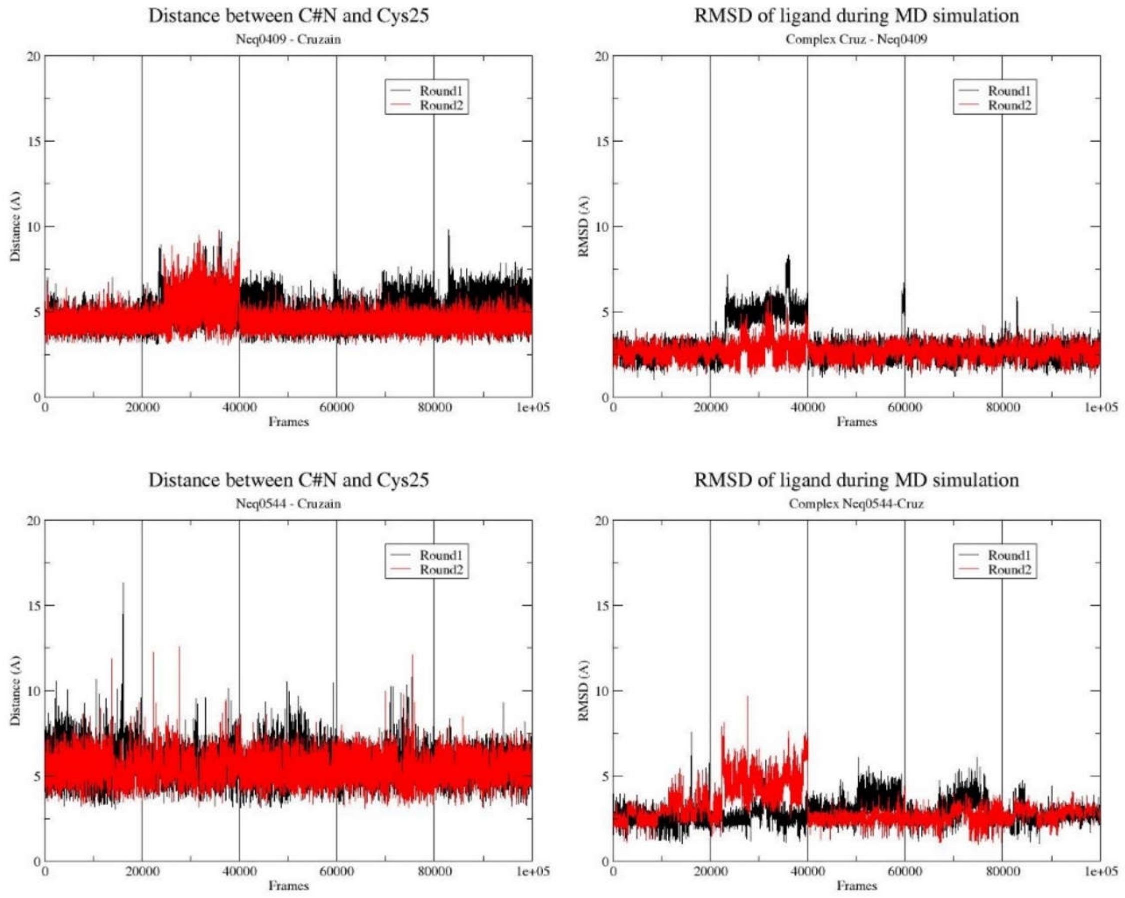

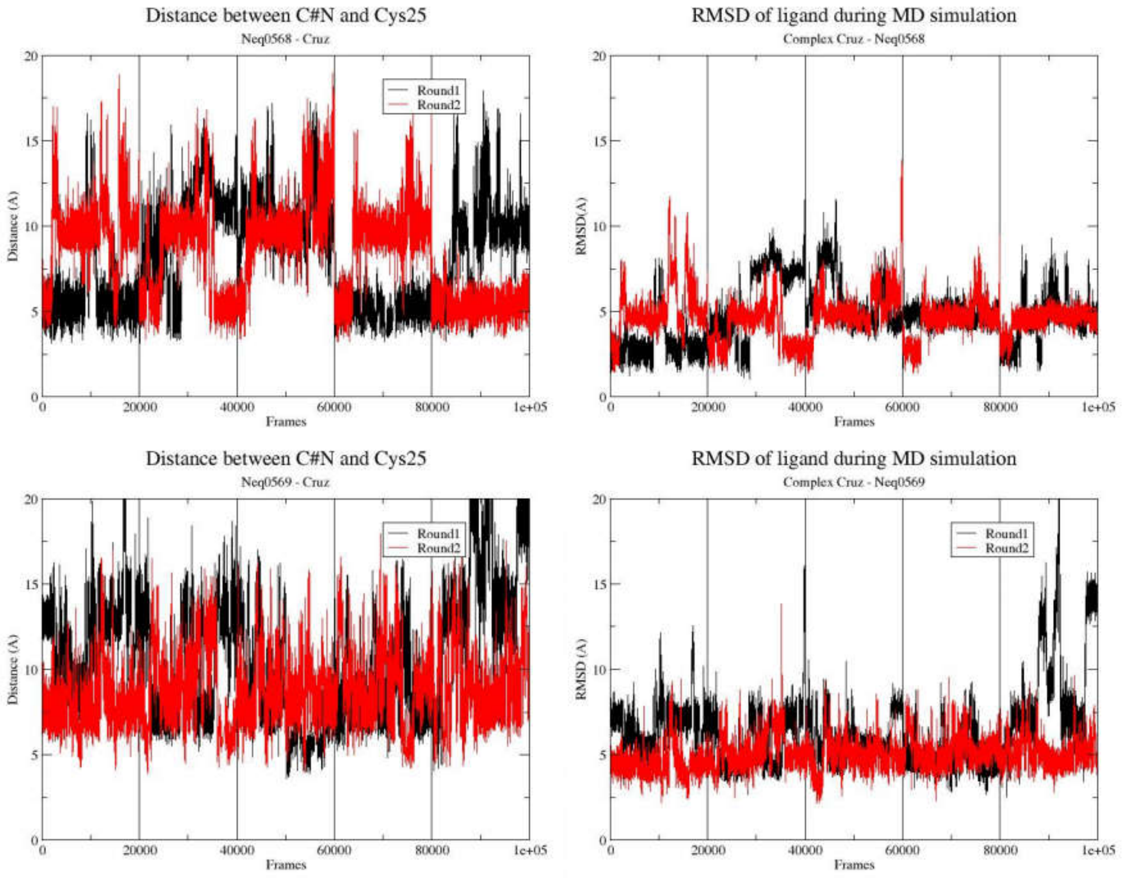

Distance between C#N and Cys25
Neq0568 - Cruz
Round1
Round2
Distance (A)
Frames
RMSD of ligand during MD simulation
Complex Cruz - Neq0568
RMSD(A)
Frames
Distance between C#N and Cys25
Neq0569 - Cruz
Round1
Round2
Distance (A)
Frames
RMSD of ligand during MD simulation
Complex Cruz - Neq0569
Round1
Round2
RMSD (A)
Frames
