# Supplementary material for: Ligand-induced conformational selection predicts the selectivity of cysteine protease inhibitors
Source: PLoS One. 2019 Dec 19;14(12):e0222055. doi: 10.1371/journal.pone.0222055 (PMC6922342; doi:10.1371/journal.pone.0222055)

Figure S 7 - Binding free energy over the time of Round 1 of simulations for ligands ICR, ICK, ICL and IKR (first, second, third and fourth column respectively) complexed with Cruzain (first row), Cathepsin K (second row) and Cathepsin L (third row). Different colors represented different replicates of the same system.

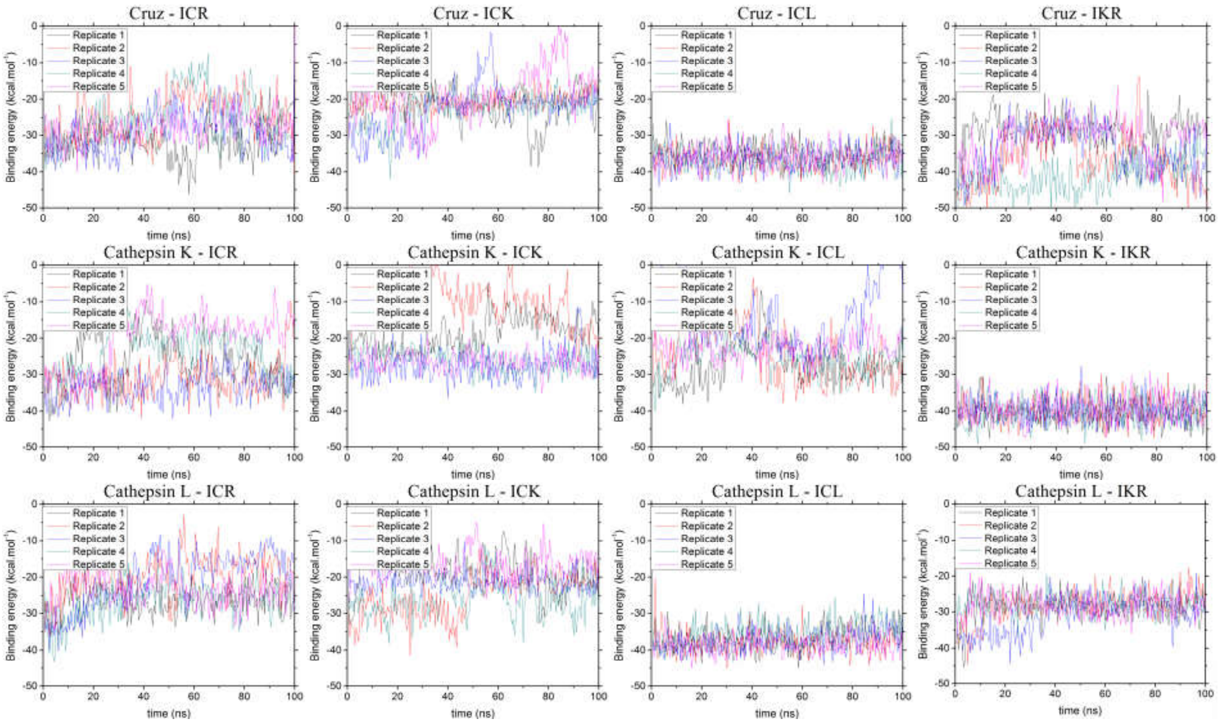

Supplement: S7 Fig — Different colors represented different replicates of the same system. (PDF) [file pone.0222055.s008.pdf]
