# Supplementary material for: Ligand-induced conformational selection predicts the selectivity of cysteine protease inhibitors
Source: PLoS One. 2019 Dec 19;14(12):e0222055. doi: 10.1371/journal.pone.0222055 (PMC6922342; doi:10.1371/journal.pone.0222055)

Figure S 9 - Projection over the first two principal components of cruzain (first row), cathepsin K (second row) and cathepsin L (third row) simulation frames in it apo form and complexed with noncovalent and covalent forms of ligand ICR (black, red and green dots, respectively).

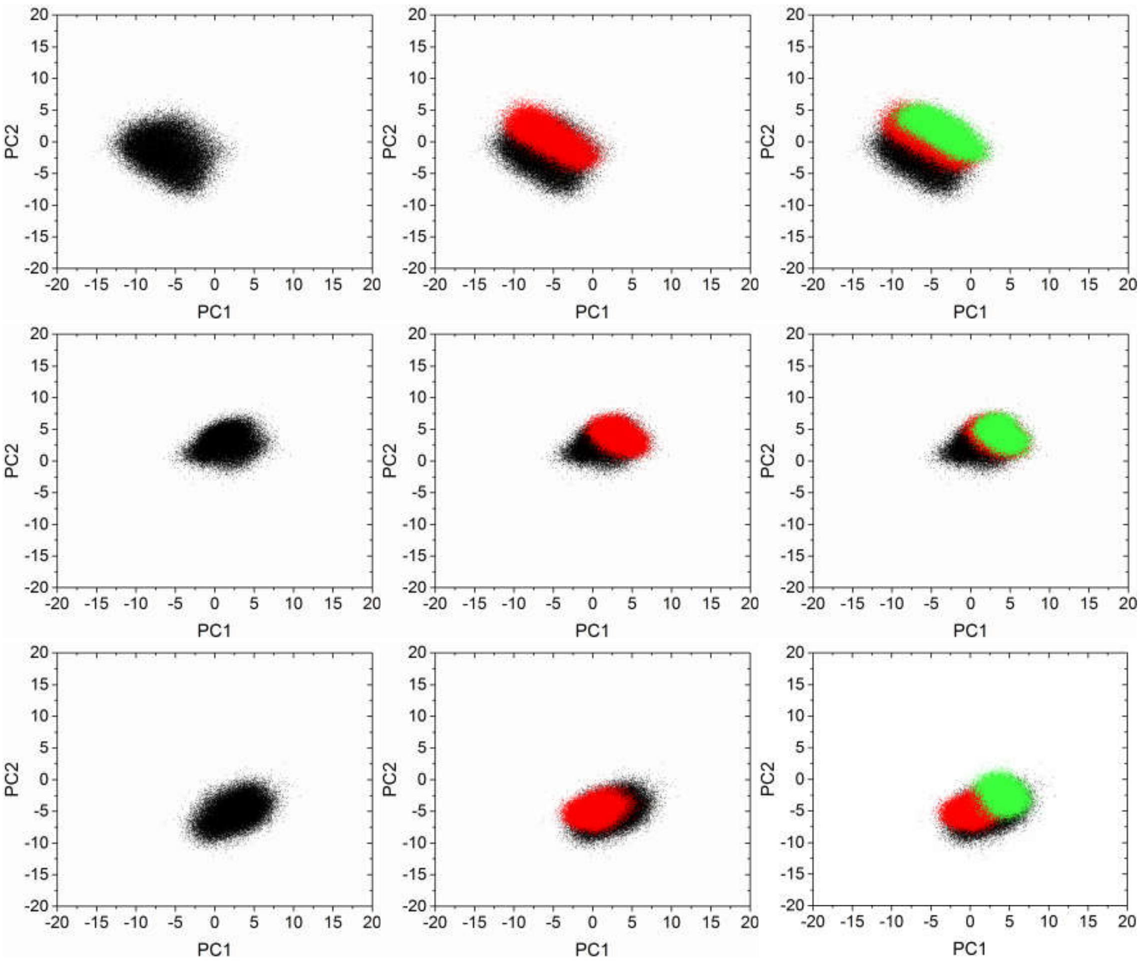

Supplement: S9 Fig — (PDF) [file pone.0222055.s010.pdf]
