# Supplementary material for: Ligand-induced conformational selection predicts the selectivity of cysteine protease inhibitors
Source: PLoS One. 2019 Dec 19;14(12):e0222055. doi: 10.1371/journal.pone.0222055 (PMC6922342; doi:10.1371/journal.pone.0222055)

Figure S 13 - Projection over the first two principal components of cruzain simulation frames in it apo (black dots) form and complexed with noncovalent (red dots) and covalent forms (green dots) of ligands Neq0409 (first row), Neq0544 (second row), Neq0569 (third row) and Neq0568 (fourth row).

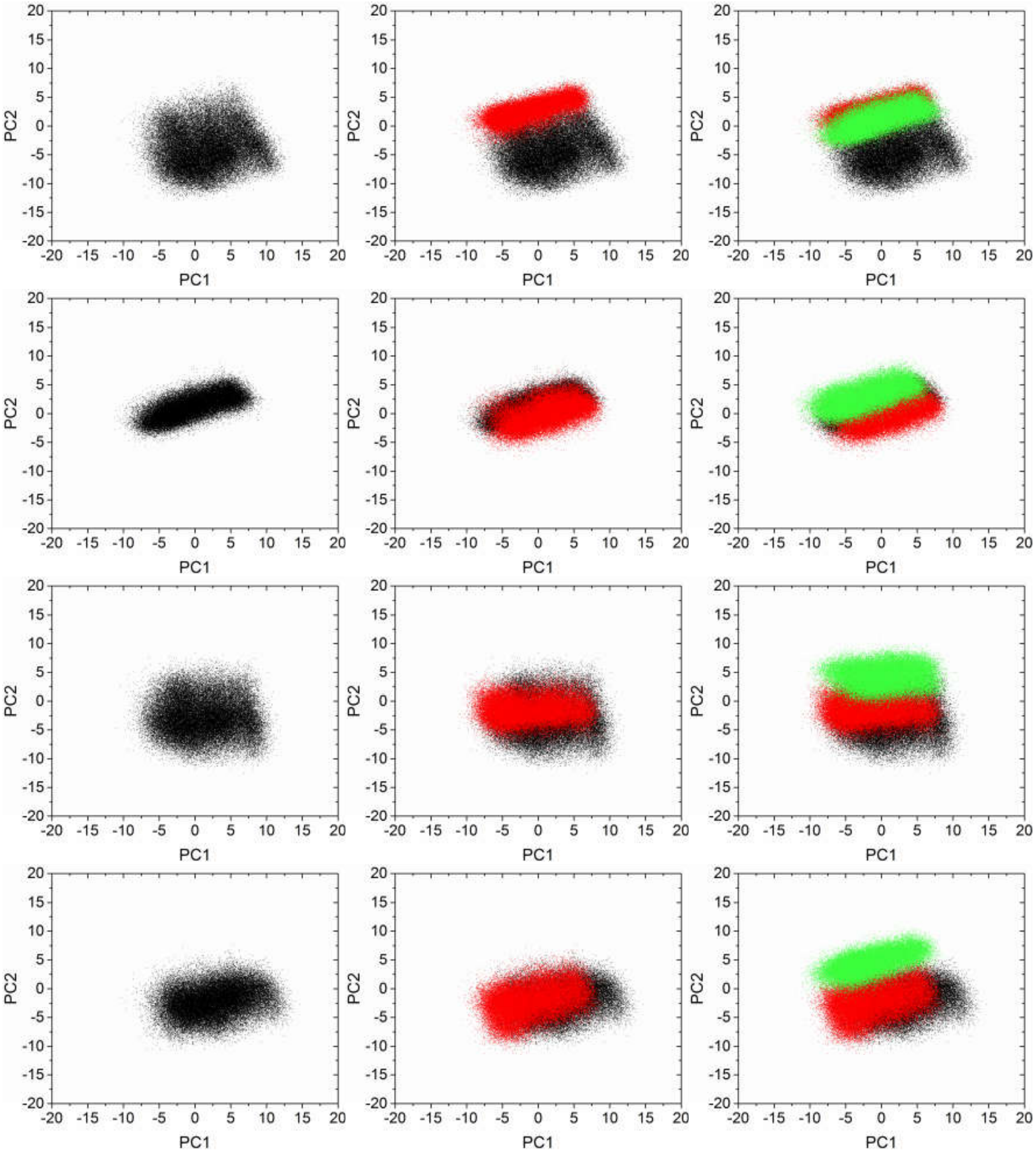

Supplement: S13 Fig — (PDF) [file pone.0222055.s014.pdf]
