# Supplementary material for: Ligand-induced conformational selection predicts the selectivity of cysteine protease inhibitors
Source: PLoS One. 2019 Dec 19;14(12):e0222055. doi: 10.1371/journal.pone.0222055 (PMC6922342; doi:10.1371/journal.pone.0222055)
Supplement: Attachment — Submitted filename: Response to Reviewers.docx [file pone.0222055.s015.docx]

PONE-D-19-23070
Ligand-induced Conformational Selection Predicts the Selectivity of Cysteine Protease Inhibitors
PLOS ONE

Dear Dr. Salsbury,

We thank you and the reviewers for your guidance. Below we detail our responses to each of the issues raised, and hope that you find this satisfactory.

Regards,

Charlie Laughton

Journal Requirements:

When submitting your revision, we need you to address these additional requirements.

1. Please ensure that your manuscript meets PLOS ONE's style requirements, including those for file naming. The PLOS ONE style templates can be found at

<http://www.journals.plos.org/plosone/s/file?id=wjVg/PLOSOne_formatting_sample_main_body.pdf> and <http://www.journals.plos.org/plosone/s/file?id=ba62/PLOSOne_formatting_sample_title_authors_affiliations.pdf>

*Response: We have gone through the manuscript carefully, and hope now it fully matches up to PLOSOne’s style requirements.*

2. We noted that several of your references did not auto populate and instead the manuscript contains the following  "Error! Reference source not found", please replace this with the appropriate reference during your next revision.

*Response: We have corrected some references; in our local version of the manuscript these format correctly (we use Mendeley) but will double-check this during the resubmission upload process.*

3. We note that you have indicated that data from this study are available upon request. PLOS only allows data to be available upon request if there are legal or ethical restrictions on sharing data publicly. For more information on unacceptable data access restrictions, please see <http://journals.plos.org/plosone/s/data-availability#loc-unacceptable-data-access-restrictions>.

In your revised cover letter, please address the following prompts:

a) If there are ethical or legal restrictions on sharing a de-identified data set, please explain them in detail (e.g., data contain potentially sensitive information, data are owned by a third-party organization, etc.) and who has imposed them (e.g., an ethics committee). Please also provide contact information for a data access committee, ethics committee, or other institutional body to which data requests may be sent.

b) If there are no restrictions, please upload the minimal anonymized data set necessary to replicate your study findings as either Supporting Information files or to a stable, public repository and provide us with the relevant URLs, DOIs, or accession numbers. For a list of acceptable repositories, please see <http://journals.plos.org/plosone/s/data-availability#loc-recommended-repositories>.

We will update your Data Availability statement on your behalf to reflect the information you provide.

*Response: Datasets (molecular dynamics trajectory files) necessary to reproduce our study findings have been deposited in Zenodo. Here are the URLs and DOIs:*

<https://zenodo.org/record/3518308>              10.5281/zenodo.3518308
<https://zenodo.org/record/3522090>              10.5281/zenodo.3522090
<https://zenodo.org/record/3523307>              10.5281/zenodo.3523307

<https://zenodo.org/record/3523367>              10.5281/zenodo.3523367


Reviewers' comments:

Reviewer's Responses to Questions

**Comments to the Author**

1. Is the manuscript technically sound, and do the data support the conclusions?

The manuscript must describe a technically sound piece of scientific research with data that supports the conclusions. Experiments must have been conducted rigorously, with appropriate controls, replication, and sample sizes. The conclusions must be drawn appropriately based on the data presented. 

Reviewer #1: Yes

Reviewer #2: Yes

*Response: no response required*

2. Has the statistical analysis been performed appropriately and rigorously? 

Reviewer #1: Yes

Reviewer #2: Yes

*Response: no response required*

3. Have the authors made all data underlying the findings in their manuscript fully available?

The [PLOS Data policy](http://www.plosone.org/static/policies.action#sharing) requires authors to make all data underlying the findings described in their manuscript fully available without restriction, with rare exception (please refer to the Data Availability Statement in the manuscript PDF file). The data should be provided as part of the manuscript or its supporting information, or deposited to a public repository. For example, in addition to summary statistics, the data points behind means, medians and variance measures should be available. If there are restrictions on publicly sharing data—e.g. participant privacy or use of data from a third party—those must be specified.

Reviewer #1: Yes

Reviewer #2: No

*Response: see above – datasets are now deposited at Zenodo*

4. Is the manuscript presented in an intelligible fashion and written in standard English?

PLOS ONE does not copyedit accepted manuscripts, so the language in submitted articles must be clear, correct, and unambiguous. Any typographical or grammatical errors should be corrected at revision, so please note any specific errors here.

Reviewer #1: Yes

Reviewer #2: Yes

*Response: no response required*

5. Review Comments to the Author

Please use the space provided to explain your answers to the questions above. You may also include additional comments for the author, including concerns about dual publication, research ethics, or publication ethics. (Please upload your review as an attachment if it exceeds 20,000 characters)

Reviewer #1: Dear Editor, dear authors,

the submitted manuscript “Ligand-induced Conformational Selection Predicts the Selectivity of Cysteine Protease Inhibitors“ describes a method to identify potent inhibitors for a cysteine protease and to predict the selectivity of the respective inhibitor. Rodrigues Sartori and colleagues nicely show that potent nitril inhibitors typically bind to their target protease via conformational selection.

Overall the study is conclusive, but some minor issues need to be addressed before publication:

(1) The authors mention that ICK is a weak cathepsin L inhibitor because it shows a high ligand RMSD during their simulations. High RMSD means the ligand tends to leave the active site more easily. Can this RMSD also be used to access / categorize the potency of cysteine protease inhibitors? Why is it not included in the current decision tree? To me this would seem to be a straight forward approach.

*Response: We address this on page 13 of the manuscript in the section “Structural Analysis of Molecular Dynamics Simulations”. We show that while it is true that in some cases (e.g. ICK) high ligand RMSD and low affinity/potency correlate, they do not always do so. This is why we did not use this in the decision tree. We have added a sentence at the end of this section to clarify this.*

(2) Nitrile inhibitors are covalent-reversible inhibitors. I would assume that potency of such an inhibitor correlates with the stability of the covalent complex. The stability of the covalent complex is not only defined by the energy of the cross-link formation, but also by the enzyme – inhibitor environment. Can the authors comment on that? How is this included in the calculations?

*Response: This is a good point, we had not fully explained our approach. We have re-written part of the section “Binding Free Energy Calculations using the MMGBSA Method” to answer this question.*

(3) Using the method described in the manuscript, is it possible to compare different classes of inhibitors? The energy of covalent bond formation might be different. How can this be included in the calculations?

*Response: A good point, we have added a short discussion of this to the conclusions section; we suggest that a QM-derived term measuring the strength of the cross-link bond would be sufficient.*

(4) On page 11, lines 410 – 417 the authors conclude that inhibitors binding to cysteine proteases via conformational selection are in general stronger inhibitors as compared to those binding via induced fit. This conclusion is based on the observation that ICL is a weak cathepsin K inhibitor. In my opinion this is not enough evidence to draw such a general conclusion. It could as well be just a coincidence.

*Response: in fairness we only claimed the result “strengthened the hypothesis” rather than proved it, but we have altered the final part of this section to qualify our analysis further, in line with the referee’s comments.*

Overall the manuscript and the figures are clear and well-made and I believe that this report will be read with great interest by the scientific community.

Reviewer #2: This manuscript combines MD simulation, PCA analysis and decision tree to identify the activity of different compounds agsinst at papain-family proteins. The topic and protocol is interesting.
Just a few issues the author need to address:
1. The author needs to put the unit of the distance and RMSD into table 2's legend.

*Response: this has been done.*

2. In line 317, it showed "Results are presented in Error! Reference source not found., and in more detail in S9-S12 Figs 318 in the supporting information.". Author needs to fix this error.

*Response: this has been done.*

3. In order to compare, the rangle of X and Y axisis should be added in figure 2.

*Response: We have amended the legend to this figure to say that all panels are at the same magnification (range of X and Y values), and what this is (40 Angstoms x 40 Angstroms).*
